# Genotyping-by-Sequencing (GBS): A Novel, Efficient and Cost-Effective Genotyping Method for Cattle Using Next-Generation Sequencing

**DOI:** 10.1371/journal.pone.0062137

**Published:** 2013-05-17

**Authors:** Marcos De Donato, Sunday O. Peters, Sharon E. Mitchell, Tanveer Hussain, Ikhide G. Imumorin

**Affiliations:** 1 Department of Animal Science, Cornell University, Ithaca, New York, United States of America; 2 Laboratorio Genética Molecular, Instituto de Investigaciones en Biomedicina y Ciencias Aplicadas Universidad de Oriente, Cumana, Venezuela; 3 Department of Animal Science, Berry College, Mount Berry, Georgia, United States of America; 4 Institute for Genomic Diversity, Cornell University, Ithaca, New York, United States of America; 5 Institute of Biochemistry and Biotechnology, University of Veterinary and Animal Sciences, Lahore, Pakistan; Kansas State University, United States of America

## Abstract

High-throughput genotyping methods have increased the analytical power to study complex traits but high cost has remained a barrier for large scale use in animal improvement. We have adapted genotyping-by-sequencing (GBS) used in plants for genotyping 47 animals representing 7 taurine and indicine breeds of cattle from the US and Africa. Genomic DNA was digested with different enzymes, ligated to adapters containing one of 48 unique bar codes and sequenced by the Illumina HiSeq 2000. *Pst*I was the best enzyme producing 1.4 million unique reads per animal and initially identifying a total of 63,697 SNPs. After removal of SNPs with call rates of less than 70%, 51,414 SNPs were detected throughout all autosomes with an average distance of 48.1 kb, and 1,143 SNPs on the X chromosome at an average distance of 130.3 kb, as well as 191 on unmapped contigs. If we consider only the SNPs with call rates of 90% and over, we identified 39,751 on autosomes, 850 on the X chromosome and 124 on unmapped contigs. Of these SNPs, 28,843 were not tightly linked to other SNPs. Average marker density per autosome was highly correlated with chromosome size (coefficient of correlation  = −0.798, r^2^  = 0.637) with higher density in smaller chromosomes. Average SNP call rate was 86.5% for all loci, with 53.0% of the loci having call rates >90% and the average minor allele frequency being 0.212. Average observed heterozygosity ranged from 0.046–0.294 among individuals, and from 0.064–0.197 among breeds, with Brangus showing the highest diversity as expected. GBS technique is novel, flexible, sufficiently high-throughput, and capable of providing acceptable marker density for genomic selection or genome-wide association studies at roughly one third of the cost of currently available genotyping technologies.

## Introduction

Beef and dairy cattle industries are major contributors to global agriculture, providing high protein foods for human consumption and many raw materials to industry. Even though cattle production has become more efficient and industrialized, profit margins remain relatively low due to high production costs, diseases and fluctuating market prices. Maximizing resources by investing in genetically superior breeding stock is a viable strategy for increasing production and cattle industry profits [1].

The search for major genes controlling production-related quantitative traits in livestock has had limited success, primarily because single gene effects tend to be small and the number of available genetic markers are insufficient for estimating effects accurately [2]. The discovery and development of large numbers of genetic markers are therefore essential for characterization and mapping of quantitative traits in cattle [3]–[5]. Genomic selection (GS) and genome-wide association studies (GWAS) are powerful statistical procedures that correlate large amounts of genetic and phenotypic data to make predictions about genetic merit. To maximize, or speed up the selection process, GS predicts desirable phenotypes by calculating breeding values based on genotype, while GWAS uses the power of historical recombination to predict which genomic region(s) influence important economic traits. Because statistical power is dependent on using large numbers of genetic markers, both methods have been limited by the cost and availability of dense genome-wide marker data [6]. The development and use of genetic markers for genotyping has been a costly and labor intensive process that could not be easily parallelized [7]. Although high-throughput single nucleotide polymorphisms (SNPs) arrays now allow for the rapid collection of fairly inexpensive genome-wide marker data [8], [3], array technology still does not address the costly and time-consuming processes of SNP discovery, development and implementation of the assay platform.

Array-based and other SNP genotyping platforms commonly include SNPs that were previously discovered by DNA sequencing. Because these SNPs may not be geographically representative and tend to be at higher frequency than random SNPs, population genetic parameters such as diversity, population subdivision and recombination estimates may be biased [9]–[11]. The degree of ascertainment bias depends on the number of individuals in which the SNPs were originally discovered and results in a skewing of the allele frequency spectrum toward common alleles [12]. This problem has a larger effect when survey populations have high levels of diversity or population substructure due to natural or artificial selection. Ascertainment bias may also impair identification of causal mutations because disequilibrium between these and genotyped SNPs may be spurious, especially if they are rare [10].

In the dairy industry, the use of SNPs for evaluating bulls as commercial semen donors has resulted in better selection at significantly reduced cost [13]. Once the value of GS was demonstrated, dairy breeders and buyers quickly adopted this strategy to improve selection efficiency [14], [15]. Although GS should also increase profits for beef bulls and dairy females, margins may be somewhat lower than for dairy bulls because the number of progeny per animal is smaller [15]–[17]. The major challenge for implementing GS is the high cost of discovery, development and genotyping of large numbers of SNPs [18], [19]. Recent advances in DNA sequencing technologies, however, have facilitated development of cost-effective and efficient strategies that allow simultaneous SNP discovery and genotyping in multiple individuals. These methods use both the power of next-generation sequencing (NGS) to obtain massive numbers of DNA sequences from the ends of genomic restriction fragments and DNA barcoding [20] for pooling of up to 384 individuals in a single sequencing lane. Various protocols, including restriction-associated DNA (RAD) [21], diversity arrays technology (DArT) [22], complexity reduction of polymorphic sequences (CRoPS) [23] and genotyping-by-sequencing (GBS) [24] are now available for obtaining sets or subsets of genomic restriction fragments for NGS. By the use of methylation sensitive restriction enzymes, repetitive genomic regions can be avoided. Thus, lower copy regions can be targeted with two to three fold higher efficiency [25], which tremendously simplifies the challenge of computational sequence alignment problems in large genome species with high levels of genetic diversity.

GBS, originally developed for crop plants [24], is a simple, reproducible, highly multiplexed approach based on the Illumina^®^ sequencing platform. The method is suitable for population studies, germplasm characterization, genetic improvement and trait mapping in a variety of diverse organisms. The major advantages over other protocols are both technical simplicity [7] and that informatics pipelines are publicly available [24]. Here, we report the adaptation of GBS for SNP genotyping in cattle. In the future we hope to use the SNPs discovered by this method for genomic selection, genome wide association studies (GWAS), and genetic characterization of populations or breeds of cattle and other livestock.

## Materials and Methods

### DNA samples and enzyme selection

Blood samples were collected from 47 unrelated animals (except for Brangus): 6 Holstein, 6 Angus, 3 Hereford and 27 Brangus from the US and 2 African indicine (White Fulani) and 3 African taurine (Muturu) cattle from Nigeria. Samples came from animals slaughtered in commercial meat plants in the US and Nigeria respectively (Table S1). US samples were collected with permission from Leona Meat Plant, Inc. Troy, PA. Nigerian samples were collected from a public abattoir. DNA was extracted using the Genomic DNA-Tissue MiniPrep (Zymo Research Corp., Irvine, CA) and quantified using an intercalating dye (QuantiFluor^TM^, Promega, Madison, WI) and a plate-format fluorometer (SpectraFluor Plus, Tecan Ltd., San Jose, CA). For optimization of the GBS protocol, 200 ng of DNA for *Ape*KI and 500 ng of DNA for *Eco*T22I, *Pst*I and *Eco*T22I/*Pst*I, were digested separately for 2 hours using a ten-fold excess of enzyme and reaction conditions as specified by the enzyme manufacturer (New England Biolabs, Ipswich, MA). After ligation of appropriate adapters (adapter amounts were determined by titration as described in Elshire et al. [24], Supplementary Information) and PCR (see below), fragment size distributions of each test library were visualized using an Agilent BioAnalyzer 2100.

### Preparation of Illumina libraries for next-generation sequencing

A 48-plex GBS library comprising 47 cattle DNA samples and a negative (no DNA) control were prepared according to Elshire et al. [24]. Briefly, individual DNA samples were digested with *Pst*I and adapters were ligated. The adapters comprised a set of 48 different barcode-containing adapters and a “common” adapter. The oligonucleotide sequences of the barcode adapters were:

- 5′ ACACTCTTTCCCTACACGACGCTCTTCCGATCTxxxxTGCA and, 5′-yyyyAGATCGGAAGAGCGTCGTGTAGGGAAAGAGTGT, where “xxxx” and “yyyy” denote the barcodes TGACGCCA, CAGATA, GAAGTG, TAGCGGAT, TATTCGCAT, ATAGAT, CCGAACA, GGAAGACAT, GGCTTA, AACGCACATT, GAGCGACAT, CCTTGCCATT, GGTATA, TCTTGG, GGTGT, GGATA, CTAAGCA, ATTAT, GCGCTCA, ACTGCGAT, TTCGTT, ATATAA, TGGCAACAGA, CTCGTCG, GCCTACCT, CACCA, AATTAG, GGAACGA, ACAACT, ACTGCT, CGTGGACAGT, TGGCACAGA, TGCTT, GCAAGCCAT, CGCACCAATT, CTCGCGG, AACTGG, ATGAGCAA, CTTGA, GCGTCCT, ACCAGGA, CCACTCA, TCACGGAAG, TATCA, TAGCCAA, ATATCGCCA, CTCTA, GGTGCACATT; and their respective complements; and the common adapters were:
- 5′-AGATCGGAAGAGCGGTTCAGCAGGAATGCCGAG and 5′-CTCGGCATTCCTGCTGAACCGCTCTTCCGATCTTGCA.

Individual ligations were pooled, and purified using QIAquick PCR Purification Kit (Qiagen, Valencia, CA). Genomic fragments were then amplified in 50 µL volumes with 10 µL pooled DNA fragments, 1X Taq Master Mix (New England Biolabs), and 12.5 pmol, each, of the following primers:

- 5′-AATGATACGGCGACCACCGAGATCTACACTCTTTCCCTACACGACGCTCTTCCGATCT and
- 5′-CAAGCAGAAGACGGCATACGAGATCGGTCTCGGCATTCCTGCTGAACCGCTCTTCCGATCT.

Temperature cycling consisted of 72°C for 5 min, 98°C for 30 s followed by 18 cycles of 98°C for 30 s, 65°C for 10 s, and 72°C for 30 s, with a final extension step at 72°C for 5 min. The *Pst*I GBS library was purified again as above, and an aliquot was run on the Agilent BioAnalyzer 2100 for evaluation of fragment sizes and the presence of adapter dimers. After quantification on the Nanodrop 2000 (Thermo Scientific, Wilmington, DE) the library was sequenced on the Illumina HiSeq 2000 at the Cornell University Life Sciences Core Facility.

### DNA sequence analysis and alignments

The raw Illumina DNA sequence data (100 nt qseq files) were processed through the GBS analysis pipeline as implemented in TASSEL v3.0 (http://www.maizegenetics.net/tassel/docs/TasselPipelineGBS.pdf). To determine copy number and genomic coordinates, sequence tags were aligned to the *Bos taurus* reference genome (UMD 3.1 using the Burrows-Wheeler alignment tool (BWA) [26]. Pipeline default parameters were used for filtering the resulting table of genotypes, except that the minimum value of F, the inbreeding coefficient (mnF), and the minimum minor allele frequency (mnMAF) were both set to 0.05 (using at least 3 individuals). Further filtering was done to eliminate SNPs present in <70% of sample DNAs.

Population genetic parameters such as genetic diversity, genetic distances and heterozygosity, were calculated and the phylogenetic analysis of the animals were carried out with TASSEL (V 4.0) and MEGA 5.05 software [27]. Pair-wise average genetic distances within and among breeds were calculated based on SNPs with call rates higher than 90% using the Maximum Composite Likelihood model with uniform mutation rates.

## Results

### Library fragment size distributions

The fragment size distributions of GBS libraries from bovine genomic DNA digested with different restriction enzymes showed that discrete peaks (i.e., repetitive DNA fragments) were present at least to some extent in all libraries (figure S1). The size distribution curve was smoothest for the *Pst*I library, with the *Ape*KI in particular, and *Eco*T22I libraries contained a higher proportion of repetitive DNAs. We found that a 196 bp repetitive DNA fragment that comprised a substantial proportion of the total population of fragments was present in the *Eco*T22I/*Pst*I double digest. As might be expected from the higher number of predicted genomic restriction sites, fragments produced by *Ape*KI (recognition sequence comprises 5 bases with one degenerate site, GCWGC) tended to be slightly smaller than fragments from six-base cutters *Pst*I (CTGCAG) and *Eco*T22I (ATGCAT) (figure S1). Based on these results, we chose to sequence GBS libraries derived from *Pst*I genomic digests containing little repetitive DNA.

### Numbers of sequences and SNPs

**Figure 1:**
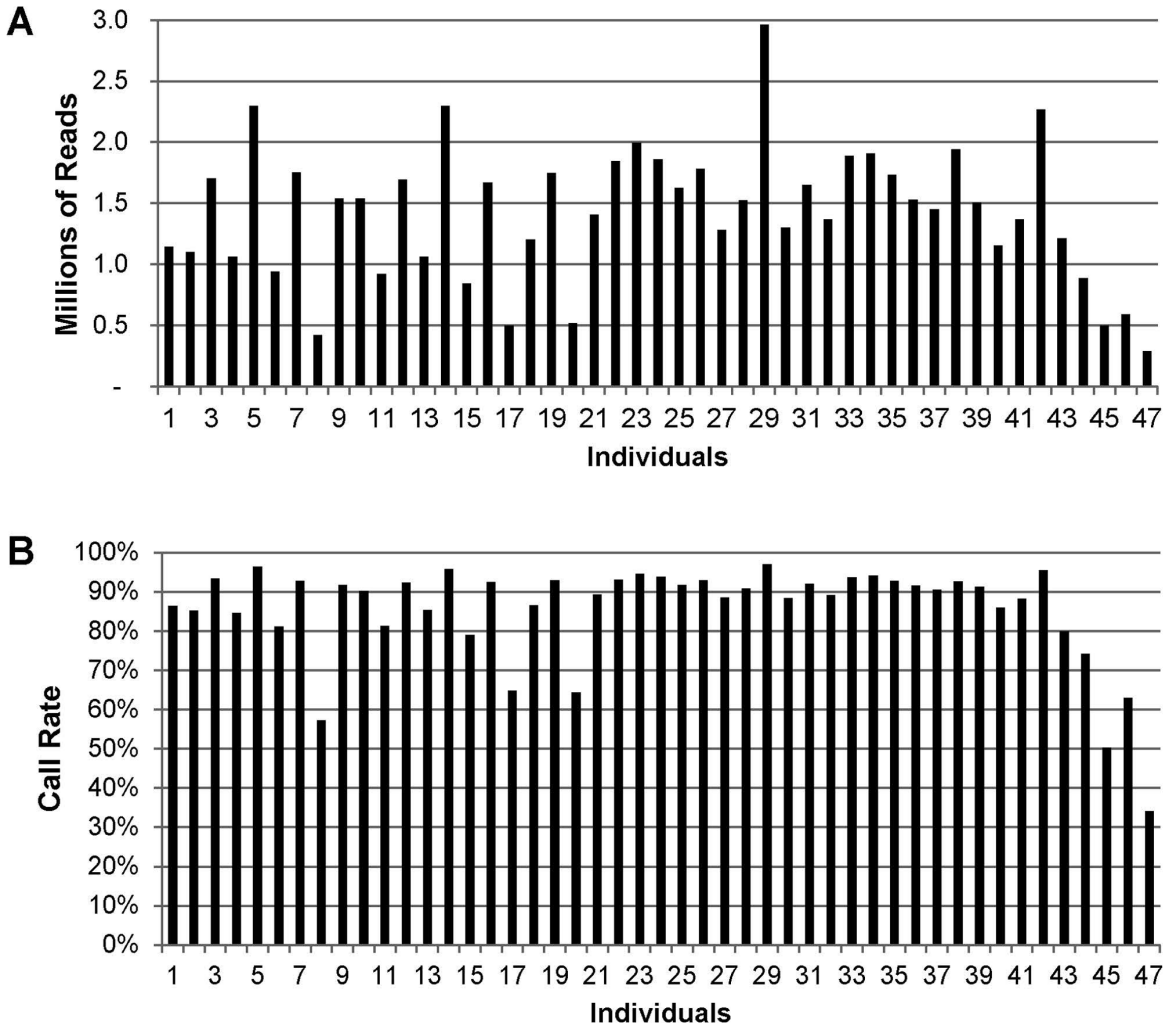
Distribution of the number of sequence reads and SNP call rates. (A) number of sequence reads in individual DNA samples. (B) Call rates of SNPs (% of total SNPs called).

Sequencing results for the 48-plex *Pst*I library showed that all 47 barcoded DNAs were represented, and that on average 1.4 million reads with a barcode and cut-site remnant were produced per animal. From these, 496,417 unique sequence tags containing 63,697 SNPs were identified. The distribution of read numbers and SNP call rates (percent of total SNPs called) in individual samples from the *Pst*I library is shown in figure 1. The coefficient of variation (CV) among read numbers per individual was 39% with 6 of the 47 samples producing less than 800,000 reads (Figure 1). After the removal of SNPs present in less than 70% of the population, the average call rate per individual was 88.2%. The 6 individuals with low read numbers also had average call rates lower than 70% (Figure 1), and one sample (with an average call rate of 34.1%) was eliminated from further analysis. The low number of reads was associated with quality of DNA, since these had lower 280/260 ratios than the other samples. In the remaining samples, the average SNP call rate across individuals was 90.1%, with 60.0% having call rates greater than 90%. If all 6 samples with low read numbers were eliminated, the average call rate would be 93.3%, and 77.2% of them with call rates greater than 90%.

**Figure 2:**
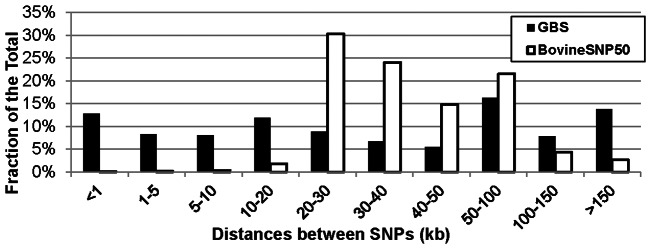
Distance between SNPs. Distribution of the distance ranges between SNPs mapped to all bovine chromosomes for GBS and for the Illumina BovineSNP50 markers.

A total of 51,414 *Pst*I-derived SNPs were identified throughout all autosomes, separated by an average distance of 48.1 kb. The X chromosome contained 1,143 SNPs, at an average distance of 130.3 kb, and 191 SNPs were located on unmapped contigs. The average number of reads per individual for the SNPs was 4.59. Only 10,953 SNPs were eliminated because of low coverage (call rates <70%). We found 15,339 (29.1%) very tightly linked SNPs, representing multiple SNPs within the same set of 64 nucleotide reads. The distribution of distances between the SNPs not tightly linked showed that 44.0% of them were <50 kb apart and with 13.8% separated by >150 kb (Figure 2). On the other hand, if we consider only the SNPs with call rates of 90% and over, we identified 39,751 on autosomes, 850 on the X chromosome and 124 on unmapped contigs for a total of 40,725 SNPs. Of those SNPs, 28,843 were not tightly linked to other SNPs.

**Figure 3:**
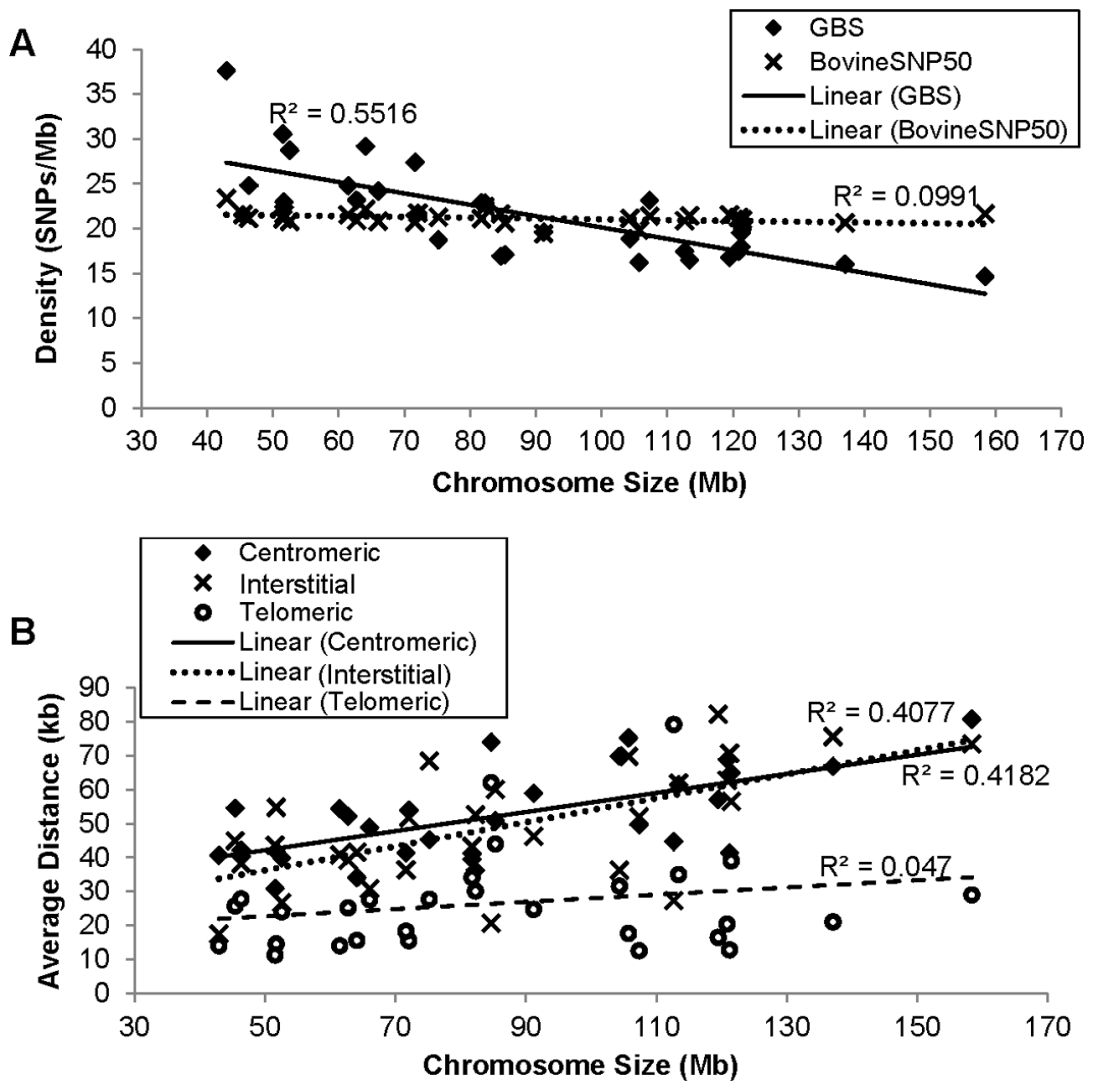
Distribution of SNPs according to chromosome size and chromosome region. (A) Average marker density per chromosome related to its size for the SNPs from the GBS and from the Illumina BovineSNP50. (B) Average distances between adjacent GBS SNPs according to chromosomal region and length. The regional SNP distances were calculated averaging the contiguous SNPs distances in the corresponding one third of each chromosomal region. R^2^ is the Pearson regression coefficient.

The average density of GBS SNPs was highly correlated with the length of the chromosomes (correlation coefficient  = −0.798, r^2^  = 0.637), with higher densities of SNPs on the smaller chromosomes (Figure 3A). In cattle, smaller chromosomes tend to be more gene rich and GBS SNP density was positively correlated with gene density (correlation coefficient  = 0.568) (Table 1). When we analyzed the distance between SNPs by chromosome region, we found that those located in interstitial regions had lower average distances than the telomeric and centromeric regions (Figure 3B). Our results showed that GBS SNPs were preferentially located either in or near gene-rich regions.

**Table 1:** Number of genes, SNPs from GBS and from the Illumina BovineSNP50 BeadChip per chromosome.

Chromosome		Number			Density (N/Mbp)		
Number	Size (Mbp)	Genes	GBS SNPs	BeadChip SNPs	Gene	GBS SNPs	BeadChip SNPs
1	158.34	1065	2322	3429	6.73	14.66	21.66
2	137.06	1038	2198	2828	7.57	16.04	20.63
3	121.43	1494	2419	2548	12.30	19.92	20.98
4	120.83	886	2110	2569	7.33	17.46	21.26
5	121.19	1525	2547	2270	12.58	21.02	18.73
6	119.46	757	2006	2574	6.34	16.79	21.55
7	112.64	1592	1966	2351	14.13	17.45	20.87
8	113.38	904	1869	2428	7.97	16.48	21.41
9	105.71	704	1716	2094	6.66	16.23	19.81
10	104.31	1219	1969	2205	11.69	18.88	21.14
11	107.31	1084	2484	2294	10.10	23,15	21.38
12	91.16	490	1788	1772	5.38	19.61	19.44
13	82.24	918	1877	1849	11.16	22.82	22.48
14	84.65	602	1432	1830	7.11	16.92	21.62
15	85.30	1357	1457	1761	15.91	17.08	20.64
16	81.72	771	1868	1725	9.43	22.86	21.11
17	75.16	725	1408	1599	9.65	18.73	21.27
18	66.00	1433	1596	1375	21.71	24.18	20.83
19	64.06	1366	1869	1419	21.32	29.18	22.15
20	72.04	395	1566	1566	5.48	21.74	21.74
21	71.60	703	1961	1482	9.82	27.39	20.70
22	61.44	627	1523	1323	10.21	24.79	21.53
23	52.53	849	1509	1092	16.16	28.73	20.79
24	62.71	374	1453	1311	5.96	23.17	20.91
25	42.90	773	1614	1003	18.02	37.62	23.38
26	51.68	469	1188	1116	9.08	22.99	21.59
27	45.41	299	977	980	6.58	21.52	21.58
28	46.31	364	1149	979	7.86	24.81	21.14
29	51.51	806	1573	1085	15.65	30.54	21.06
X	148.82	1401	1143	1169	9.41	7.68	7.86
Total	2658.9	26990	52748*	54026	10.64	19.77	20.71

Chromosome size and number of genes were obtained from the bovine assembly UMD_3.1 (http://www.ncbi.nlm.nih.gov/projects/mapview/map_search.cgi?taxid=9913).

* Includes 191 SNPs not placed on cattle chromosomes in the UMD 3.1 assembly.

### Sequence coverage depth

**Figure 4:**
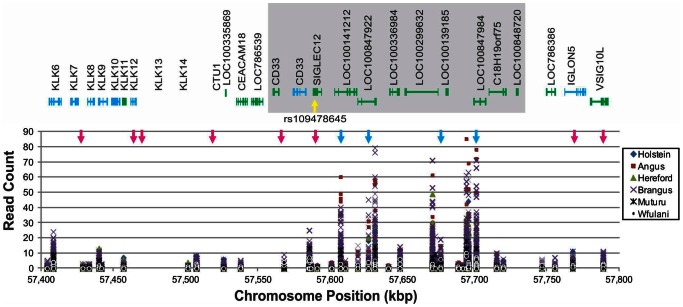
Detection of copy number variation (CNVs). Read counts in all the sequenced tags and individuals for a region on Chromosome 18 that have been reported to contain a polymorphic CNV (gray area) [28] and a SNP (yellow arrow) in the second intron of the gene SIGLEC12 that was associated with economic traits in dairy cattle [29]. Light blue arrows show the position of the SNPs found by GBS and the dark blue are the SNPs included in the Bovine SNP50 Bead Chip. Gene annotation was obtained from MapView at the NCBI database for the bovine assembly UMD_3.1.

Out of approximately 0.5 million unique tags, the average coverage depth was 2.3 reads per tag (locus). We found that 34.9% of the tags showed counts >10 in at least 1 individual. The distribution of read counts among all individuals showed specific clustering along the chromosomes (Figure S2). These clusters could reflect copy number variations (CNVs), sequencing biases, or a combination of both. To investigate whether GBS data can show the presence of CNVs, we analyzed the counts per animal for all reads (polymorphic or not) in a 0.4 Mb region of chromosome 18 that contains a polymorphic duplication [28]. Results showed a polymorphism in the number of reads for Holstein, Angus, Hereford and Brangus individuals, but not for the African breeds of Muturu and White Fulani (Figure 4).

SNPs appear to be largely evenly distributed along the chromosomes (Figure S3), but with higher densities towards the telomeres in most of the chromosomes with the highest density bias found in telomeres on chromosomes 3, 5, 6 and X. However, chromosomes 7, 14 and 25 showed regions other than the telomeres with SNP density biases. In comparison, the markers on the Illumina BovineSNP50 BeadChip as expected were distributed evenly along the chromosomes, except for some density biases on a few autosomes (e.g. chromosomes 7 and 11), as well on the X chromosome.

### Allele frequencies

**Figure 5:**
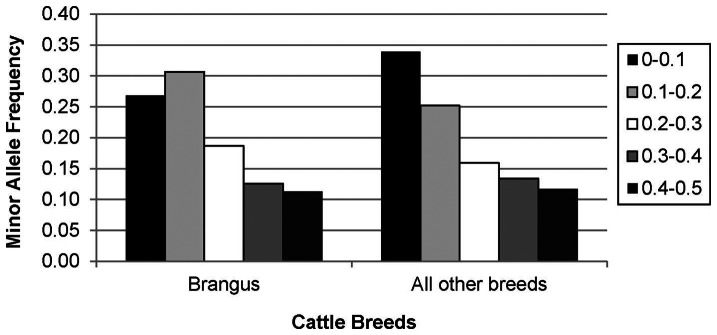
Distribution of the minor allele frequency (MAF). MAF classes in all the animals, in Brangus cattle and in all the other breeds of cattle.

The overall average minor allele frequency (MAF) for the GBS SNPs was 0.212. Brangus showed the highest MAF average of 0.215 (Table 2) and its allele frequency distribution was somewhat different compared to other breeds, with the most frequent class being MAF between 0.1-0.2 and higher frequencies for the 0.2-0.3 and 0.3–0.4 classes (Figure 5). Average observed heterozygosity (Ho) per individual ranged from 0.047 to 0.299, with the Nigerian White Fulani breed (indicine cattle) having the lowest (0.090) and the Brangus (indicine x taurine) having the highest Ho (0.195) average (Table 2). Angus animals showed the lowest genetic diversity compared to other breeds, while Muturu, in contrast, showed the highest within breed genetic diversity, although the small number of animals sampled per breed precludes any definite conclusion. The breeds that showed the lowest average distances between them were Angus and Hereford, as well as Angus and Brangus. Muturu showed the highest genetic differentiation from other breeds.

**Table 2:** Pair-wise average genetic distances among the breeds studied and the within breed distances.

Breed	Holstein	Angus	Hereford	Brangus	Muturu	Within Breed	MAF	H_O_
Holstein	–					0.064	0.133	0.127
Angus	0.041	–				0.008	0.141	0.134
Hereford	0.056	0.022	–			0.028	0.107	0.116
Brangus	0.064	0.030	0.045	–		0.052	0.203	0.201
Muturu	0.445	0.398	0.433	0.429	–	0.701	0.160	0.116

White Fulani was not analyzed because there was only one animal adequate good call rate.

H_O_: average fraction of observed heterozygous sites.

### Relationship among breeds and animals

**Figure 6:**
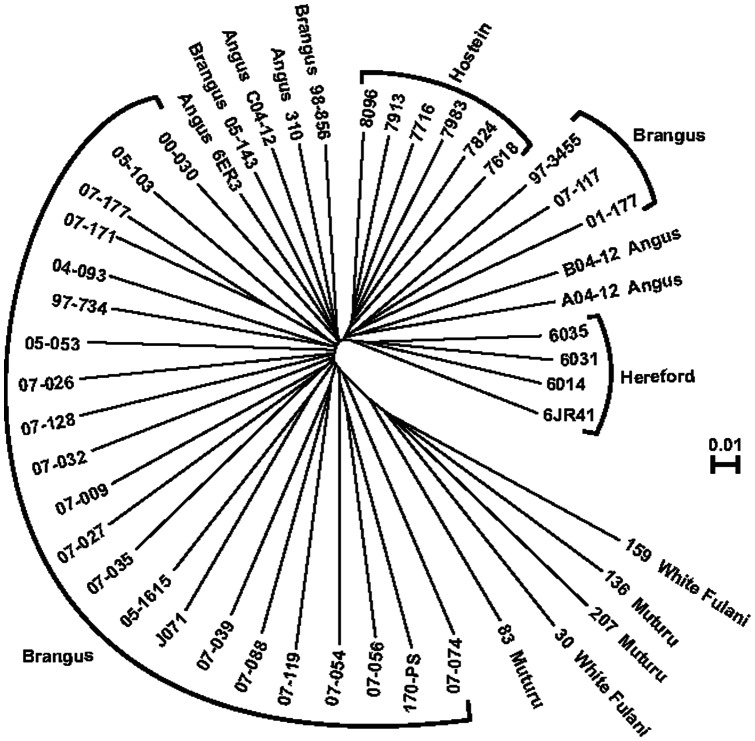
Neighbor-joining analysis showing the relationship among cattle breeds. The dendrogram was constructed using TASSEL.

A neighbor-joining analysis of the relationships among samples showed that animals usually grouped by breed (Figure 6). There was some intermixing of Angus and Brangus individuals, which probably reflects backcrossing of Brangus to the Angus progenitor. Additionally, the African breeds do not appear to be resolved and showed some degree of admixture. In Brangus, some individual relationships can be seen. For example, individuals 07–171 and 07–177 are full siblings, and the relationship among them is the closest of the group. Relationships among individuals belonging to the same herd are also evident (e.g. 07–032, 07–009, 07–027, 07–035).

## Discussion

An average MAF of 0.272 was reported in three DNA pools representing 15 Holstein lineages, 35 Angus bulls and a mixed population of minor beef breeds (at least two bulls each of Charolais, Gelbvieh, Hereford, Limousin, Red Angus and Simmental), using reduced representation libraries (RRLs) [11], while an average of 0.248 was reported in a population of 576 individuals from 21 cattle breeds, using Illumina BovineSNP50 BeadChip [3]. Both of those averages were somewhat higher than the average we found in our study. However, since SNPs derived by GBS or other *de-novo* sequencing methodologies are developed as they are sequenced, they can be used in any population without ascertainment bias [24], as shown in maize when comparing GBS data with the one obtained using SNP chip containing 55,000 markers [30]. In addition, ascertainment bias can affect allele frequencies.

Copy number variations (CNVs) have been identified in human and other mammalian genomes and are now considered a source of heritable variation in complex traits [31]. Studies in cattle have been able to associate specific CNVs to dairy traits [28] and parasite resistance/susceptibility [32]. A study in dairy cattle identified a SNP in the second intron of the *SIGLEC12* gene on chromosome 18 (rs109478645) associated with sire and daughter calving ease, strength, stature, body depth, and rump width [29]. A polymorphic CNV in the same chromosome region was located in Holstein cattle [28], and this CNV is consistent with differences in read counts for this region in our study. This indicates that GBS may be able to detect segment gains, although it will not detect segment losses at this scale because they will look like low coverage SNPs and will probably be filtered out of the data set. Higher coverage depth may allow detection of CNV contractions but the cost of genotyping will likely increase by 40–50% per additional sequencing run.

We can estimate the total number of polymorphic sites found from the total number of SNPs divided by the total number of tags, which in this case is 2.88 sites per kilo base pairs. Although this estimate should be lower than the actual value since the number of individuals and breeds investigated is small, it is higher than the estimate for the human genome of 2.19 (7 million SNPs in a genome of 3.2 billion bases) [33]. Extrapolating from the 22.1 Mb of sequence obtained by GBS and the total number of SNPs identified (63,697), the number of SNPs in the bovine genome should be more than 8.6 million. A substantially lower estimate of 1.25 SNPs per kilobase pairs can be deduced from the RRL data reported by Van Tassell et al. [11] (62,042 total SNPs from 49,492,755 bases of sequence). GBS data has shown higher SNP densities in telomeric and some centromeric regions, which is consistent with previous reports from whole genome sequencing [34]. These studies found that greater SNP densities in or near centromeres and telomeres were not explained by differences in read depth across the genome. They also found that there was a slight increase in the number of SNPs in smaller chromosomes and that the SNPs were not evenly distributed in the genome but their density was higher in regions closer to genes. GBS showed a high correlation between densities of genes and SNPs. This could be related to the fact that *Pst*I is methylation-sensitive possibly resulting in a bias for incorporation of under-methylated regions of the genome, which are also associated with high gene density.

**Figure 7:**
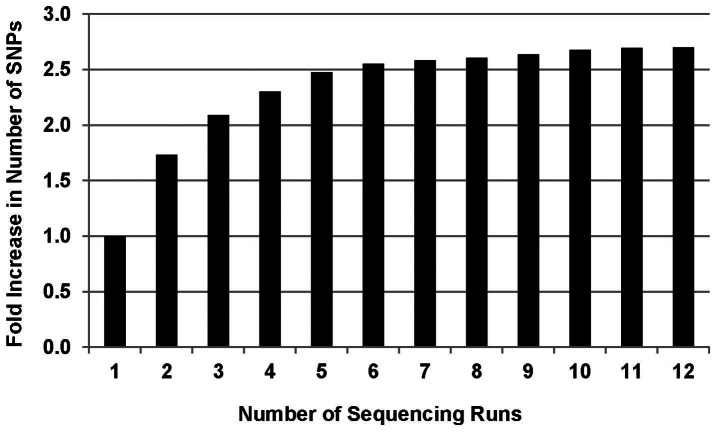
Increase in SNP numbers resulting from multiple sequencing runs. These data correspond to SNPs detected on chromosome 1 in two lines of *Sorghum bicolor* (Mitchell SE, unpublished) taken from another study. Here, 384 samples per library were run in a single lane. Since the sorghum genome (730 Mbp) [35] is approximately ¼ of the size of the cattle genome, this sorghum 384-plex is comparable to 96-samples per lane in cattle, which have an estimated genome size of 3000 Mbp [36].

An analysis of unpublished data from repeated sequencing of *Sorghum bicolor Ape*KI libraries (Mitchell SE, unpublished) has shown that SNP numbers increase with each sequencing run and this effect is especially pronounced during the first few runs (Figure 7). Thus, running a GBS library 4 times should increase the number of SNPs identified about 2.3 fold and the increased sequence depth per locus (DNA fragment) will increase statistical support both for identifying variants and calling heterozygotes. However, the number of GBS SNPs identified in cattle at an average distance of around 50 kb is less than one third of the mean LD block length, which is estimated in Holsteins to be 164±117 kb [37]. Therefore, it should be possible to detect most of the LD blocks associated with any trait in a single sequencing run in that breed.

GBS has been shown here to be an efficient and cost-effective method for the simultaneous discovery and genotyping of large numbers of SNPs in cattle (over 51, 000 SNPs) with high quality at a cost of less than $30 per individual with multiplexing of 96 samples per lane. SNP genotyping by using the BovineSNP50 BeadChips surveys a similar number of SNPs (54,609) but at >3X higher cost per sample. An additional benefit of GBS is that depending on the budget, the number of SNPs and sequence coverage per SNP locus can be increased by running the same GBS libraries in additional sequencing lanes. For the same cost as chip genotyping, four additional single end sequencing runs can be performed, potentially increasing the number of SNPs with high call rate by an estimated 2.5 fold (>130,000 SNPs with call rate >70% and >72,000 SNPs with call rate >90%). This method also has great potential for application in other domestic genomes such as sheep, goat, and buffalo whose reference sequences are either being developed or for which additional breeds are yet to be fully sequenced. Even though the availability of reference genomes is very useful to eliminate repetitive sequences, GBS can be used without a reference genome, by either using consensus sequences of reads as the reference or using the tags simply as dominant markers [24]. In addition, imputation of missing data using GBS markers can be done with extremely high accuracy, with a reference genome in biparental mapping populations, allowing for low coverage of the offspring at an even lower cost, after genotyping parents and grandparents at high coverage [24], [38].

## Supporting Information

Supplemental Figure S1 **Fragment size distribution of the GBS libraries.** Fragment size distribution of GBS libraries made with a single DNA sample using three restriction enzymes, separately, and one double digest. Libraries were run on an Agilent BioAnalyzer 2100. The x-axis represents elution time and the y-axis shows fluorescence units. Numbers above hatch marks on the x-axis indicate fragment size in bp. Tall peaks at 15 and 1500 bp are size standards.
(PDF)

Supplemental Figure S2 **Distribution of cattle GBS SNPs by chromosome.** Location along each of the 30 bovine chromosomes of the SNPs from the GBS (thin line) and the Illumina BovineSNP50 (thick, more straight line). The thin diagonal, straight line represents a distribution with homogeneous distribution of SNPs along the chromosome. The Y axis is the chromosome location in Mbp and the X axis is the order of the markers.
(PDF)

Supplemental Table S1 **Animals used in this study**. Breeds and origin of the animals used in this study.
(DOCX)
