# Supplementary figures and images for: Genotyping-by-Sequencing (GBS): A Novel, Efficient and Cost-Effective Genotyping Method for Cattle Using Next-Generation Sequencing
Source: PLoS One. 2013 May 17;8(5):e62137. doi: 10.1371/journal.pone.0062137 (PMC3656875; doi:10.1371/journal.pone.0062137)

Fluorescence

*ApeKI*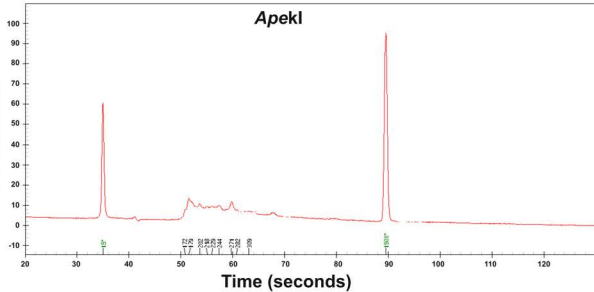

Fluorescence

*PstI*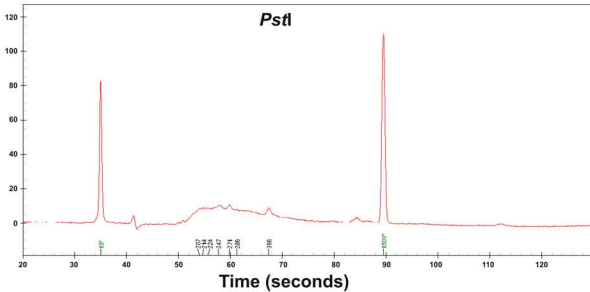

Fluorescence

*EcoT22I*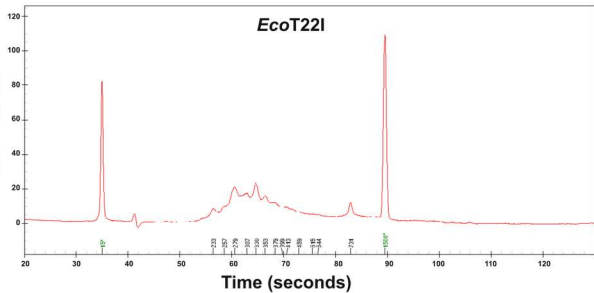

Fluorescence

*EcoT22I-PstI*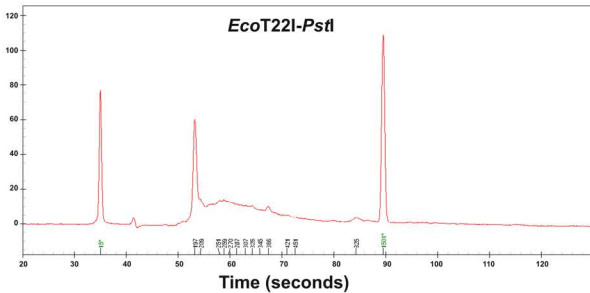

Supplement: Supplemental Figure S1 — Fragment size distribution of the GBS libraries. Fragment size distribution of GBS libraries made with a single DNA sample using three restriction enzymes, separately, and one double digest. Libraries were run on an Agilent BioAnalyzer 2100. The x-axis represents elution time and the y-axis shows fluorescence units. Numbers above hatch marks on the x-axis indicate fragment size in bp. Tall peaks at 15 and 1500 bp are size standards. (PDF) [file pone.0062137.s001.pdf]

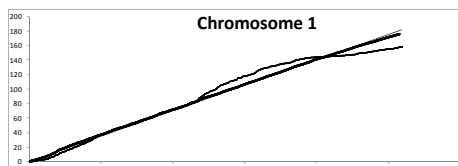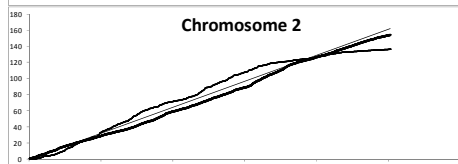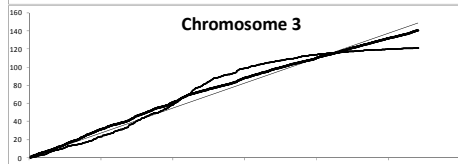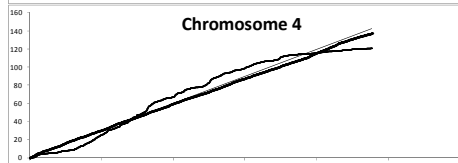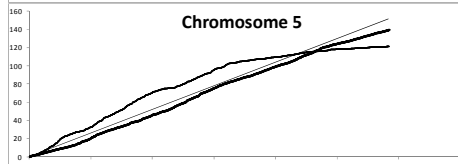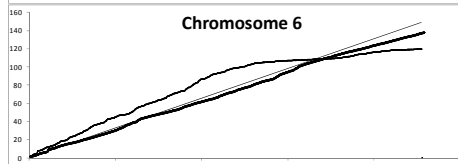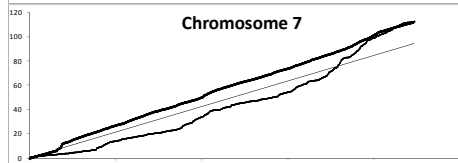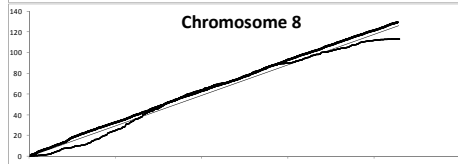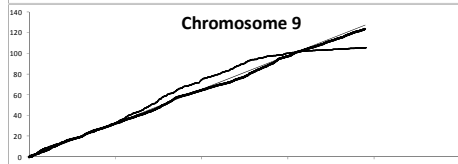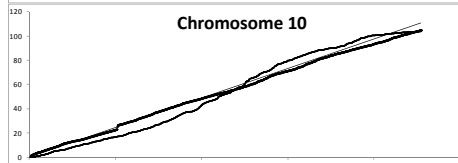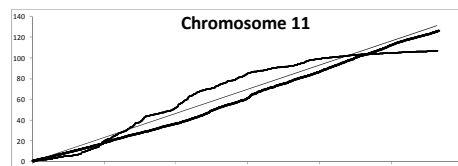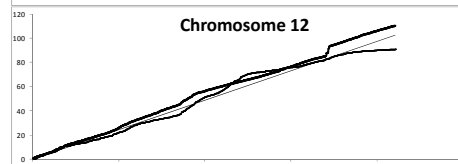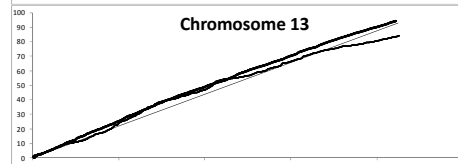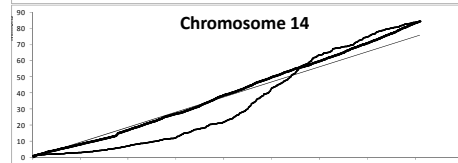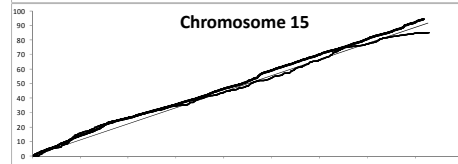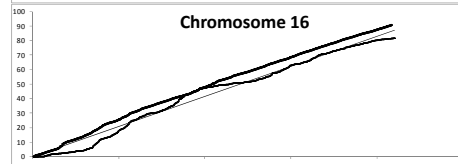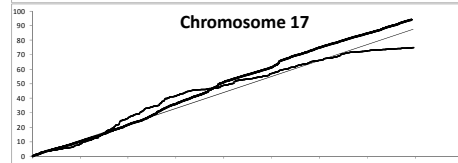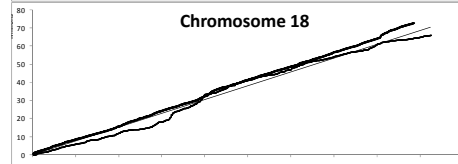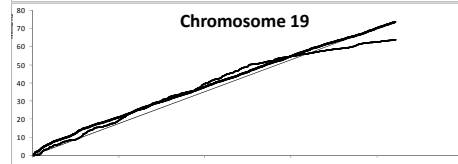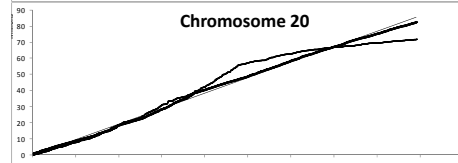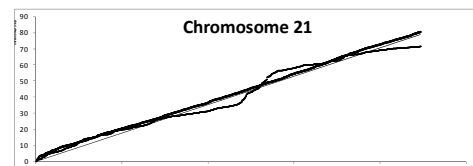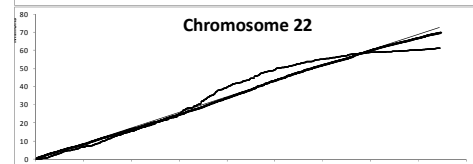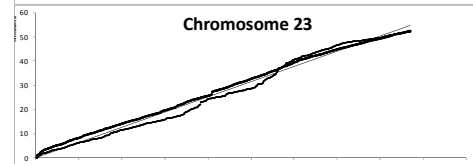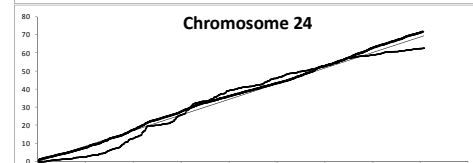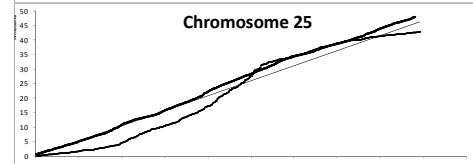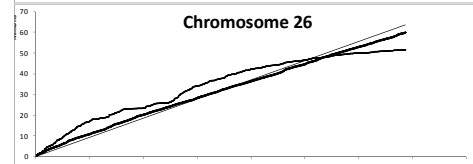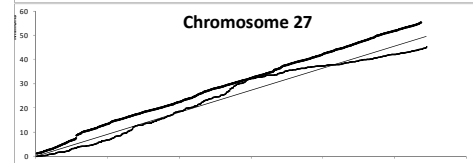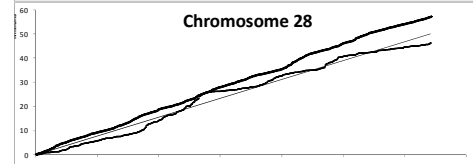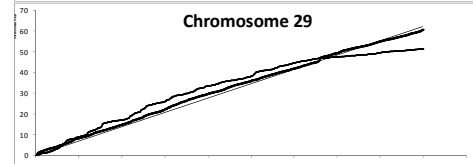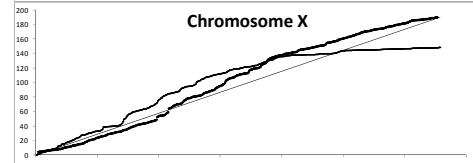

Supplement: Supplemental Figure S2 — Distribution of cattle GBS SNPs by chromosome. Location along each of the 30 bovine chromosomes of the SNPs from the GBS (thin line) and the Illumina BovineSNP50 (thick, more straight line). The thin diagonal, straight line represents a distribution with homogeneous distribution of SNPs along the chromosome. The Y axis is the chromosome location in Mbp and the X axis is the order of the markers. (PDF) [file pone.0062137.s002.pdf]
