# Supplementary material for: Genotyping-by-Sequencing (GBS): A Novel, Efficient and Cost-Effective Genotyping Method for Cattle Using Next-Generation Sequencing
Source: PLoS One. 2013 May 17;8(5):e62137. doi: 10.1371/journal.pone.0062137 (PMC3656875; doi:10.1371/journal.pone.0062137)
Supplement: Supplemental Table S1 — Animals used in this study. Breeds and origin of the animals used in this study. (DOCX) [file pone.0062137.s003.docx]

Supplemental Table S1. Animals used in this study.

| Sample Number | Sample Code | Type of Cattle | Breed | Country |
| --- | --- | --- | --- | --- |
| 1 | 7618 | Taurine | Holstein | USA |
| 2 | 7716 | Taurine | Holstein | USA |
| 3 | 7824 | Taurine | Holstein | USA |
| 4 | 7913 | Taurine | Holstein | USA |
| 5 | 7983 | Taurine | Holstein | USA |
| 6 | 8096 | Taurine | Holstein | USA |
| 7 | 310 | Taurine | Angus | USA |
| 8 | 6ER3 | Taurine | Angus | USA |
| 9 | 6JR41 | Taurine | Angus | USA |
| 10 | 0411A | Taurine | Angus | USA |
| 11 | 0411B | Taurine | Angus | USA |
| 12 | 0411C | Taurine | Angus | USA |
| 13 | 6014 | Taurine | Hereford | USA |
| 14 | 6031 | Taurine | Hereford | USA |
| 15 | 6035 | Taurine | Hereford | USA |
| 16 | 01-177 | Taurine x indicine | Brangus | USA |
| 17 | 04-093 | Taurine x indicine | Brangus | USA |
| 18 | 05-053 | Taurine x indicine | Brangus | USA |
| 19 | 05-103 | Taurine x indicine | Brangus | USA |
| 20 | 05-143 | Taurine x indicine | Brangus | USA |
| 21 | 05-1615 | Taurine x indicine | Brangus | USA |
| 22 | 07-009 | Taurine x indicine | Brangus | USA |
| 23 | 07-026 | Taurine x indicine | Brangus | USA |
| 24 | 07-027 | Taurine x indicine | Brangus | USA |
| 25 | 07-032 | Taurine x indicine | Brangus | USA |
| 26 | 07-039 | Taurine x indicine | Brangus | USA |
| 27 | 07-054 | Taurine x indicine | Brangus | USA |
| 28 | 07-056 | Taurine x indicine | Brangus | USA |
| 29 | 07-074 | Taurine x indicine | Brangus | USA |
| 30 | 07-088 | Taurine x indicine | Brangus | USA |
| 31 | 07-119 | Taurine x indicine | Brangus | USA |
| 32 | 07-128 | Taurine x indicine | Brangus | USA |
| 33 | 07-171 | Taurine x indicine | Brangus | USA |
| 34 | 07-171 | Taurine x indicine | Brangus | USA |
| 35 | 170-PS | Taurine x indicine | Brangus | USA |
| 36 | 97-734 | Taurine x indicine | Brangus | USA |
| 37 | 98-856 | Taurine x indicine | Brangus | USA |
| 38 | J071 | Taurine x indicine | Brangus | USA |
| 39 | 00-030 | Taurine x indicine | Brangus | USA |
| 40 | 07-035 | Taurine x indicine | Brangus | USA |
| 41 | 97-3455 | Taurine x indicine | Brangus | USA |
| 42 | 07-117 | Taurine x indicine | Brangus | USA |
| 43 | 83 | African | Muturu | Nigeria |
| 44 | 207 | African | Muturu | Nigeria |
| 45 | 136 | African | Muturu | Nigeria |
| 46 | 30 | Indicine | Wfulani | Nigeria |
| 47 | 159 | Indicine | Wfulani | Nigeria |
